# Social Distance Evaluation in Human Parietal Cortex

**DOI:** 10.1371/journal.pone.0004360

**Published:** 2009-02-10

**Authors:** Yoshinori Yamakawa, Ryota Kanai, Michikazu Matsumura, Eiichi Naito

**Affiliations:** 1 Graduate School of Informatics, Kyoto University, Sakyo-ku, Kyoto, Japan; 2 The Japan Society for the Promotion of Science, Tokyo, Japan; 3 ATR Computational Neuroscience Laboratories, Kyoto, Japan; 4 NEC Corporation Corporate Planning Division, Tokyo, Japan; 5 Institute of Cognitive Neuroscience, University College London, London, United Kingdom; 6 Graduate School of Human and Environmental Studies, Kyoto University, Sakyo-ku, Kyoto, Japan; 7 National Institute of Information and Communication Technology, Research Department 1, Kobe Advanced ICT Research Center, Biophysical ICT Group, Kyoto, Japan; Chiba University Center for Forensic Mental Health, Japan

## Abstract

Across cultures, social relationships are often thought of, described, and acted out in terms of physical space (e.g. “close friends” “high lord”). Does this cognitive mapping of social concepts arise from shared brain resources for processing social and physical relationships? Using fMRI, we found that the tasks of evaluating social compatibility and of evaluating physical distances engage a common brain substrate in the parietal cortex. The present study shows the possibility of an analytic brain mechanism to process and represent complex networks of social relationships. Given parietal cortex's known role in constructing egocentric maps of physical space, our present findings may help to explain the linguistic, psychological and behavioural links between social and physical space.

## Introduction

In our highly complex human society, social intelligence is essential for interacting with other agents [1], [2]. One of the key elements of social intelligence is the ability to assess social relationships between oneself and others [3], [4], as it influences our socioeconomic behaviours [5], [6], [7]. Across cultures, the nature of interpersonal relationships are often thought of, described and acted out in terms of physical space (e.g. “close friends” or “distant relatives”) [8], [9].

It has been widely observed that there is a tendency for people to cognitively map social distances onto physical space, giving rise to psychological tools such as sandplay therapy and sociograms. Moreover, social distances are also acted out in our natural behaviours, such as our tendency to regulate “personal space” based on the degree of social connection with others [10], [11]. However, whether the connection between spatial concepts and social concepts in linguistics, psychology and behaviour is just a convenient metaphor or has biological roots in the brain remains unexplored. One intriguing possibility is that the connection between the mental representations of social relationships and those of physical space is based on common neural substrates in the brain [12]. In particular, since the parietal cortex is known to be involved in the self-referential operations that convert the spatial information of external objects into self-centred (i.e. egocentric) coordinates for action behaviour [13]–[17], the common origin hypothesis predicts that the parietal cortex should also be engaged in social distance judgments, when a self-referential process is required [18]. If the parietal cortex indeed performs analogous operations in social space, such a self-referential mapping of social distance would be an efficient manner of organizing complex social information to guide interactions with others.

**Figure 1:**
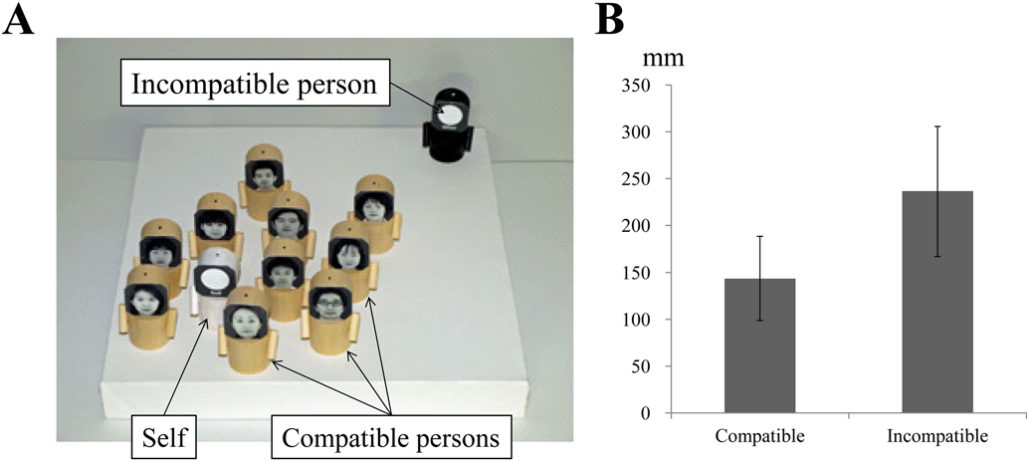
Results of a doll-arrangement task. (A) A typical arrangement by a participant, with compatible dolls placed close to the self doll. (B) Average distances between the self and incompatible dolls for all participants. The average distance between the self and compatible dolls across participants was significantly (physically) shorter than that between the self and incompatible dolls. Error bars indicate the standard deviation.

In the present study, first, we investigate whether people symbolically organize social relationships on a ‘distance’ scale when estimating social compatibility with other agents. In this psychophysical experiment, fifteen participants performed a doll-arrangement task (Figure 1). First, they had to place a doll representing self and another doll representing an incompatible person wherever they liked on a stage, and next the participants were requested to select dolls representing compatible persons based on their facial pictures and to spatially arrange on the stage wherever they liked. By measuring physical distances between a self-doll and other dolls, we may know if people represent social relationships on a ‘distance’ scale when estimating social compatibility with other agents.

**Figure 2:**
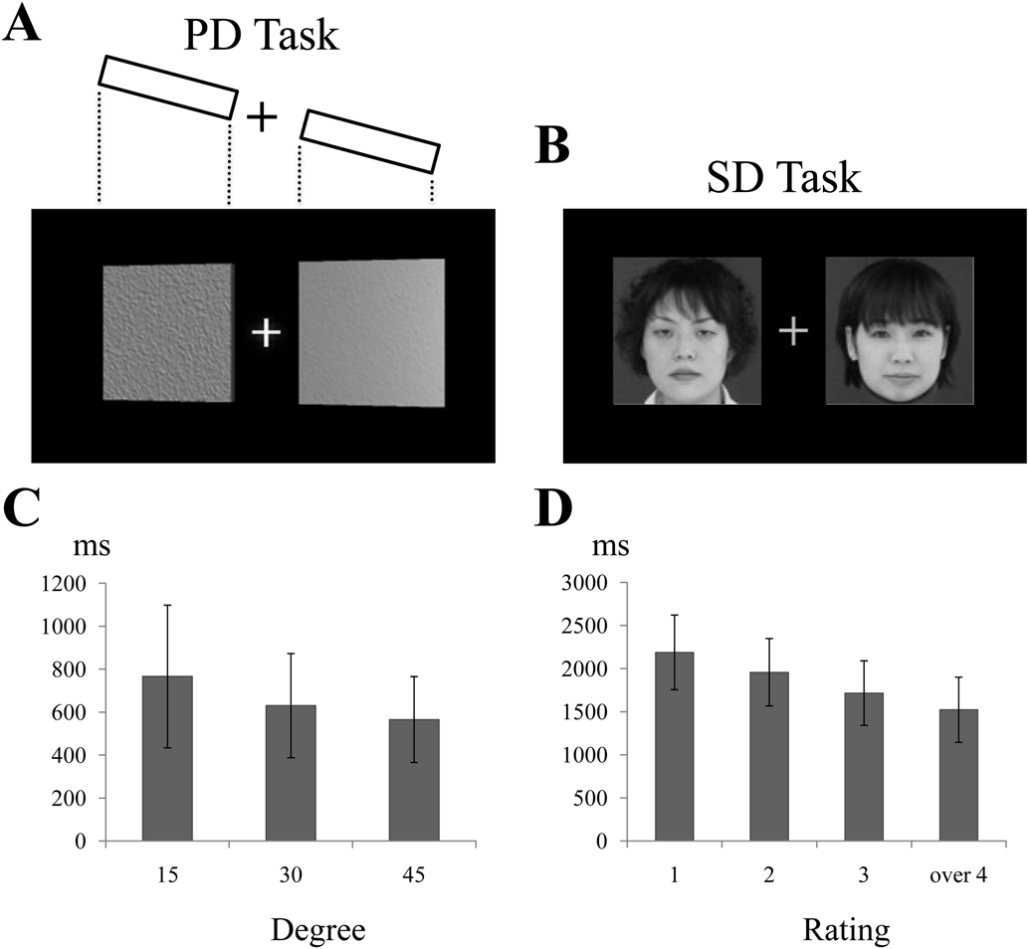
Examples of stimuli used in the PD and SD tasks and the average results. (A) Stimulus for PD task. Texture and lighting cues on the pair of panels reflect a physical arrangement. Trials varied in terms of the angle of the arrangement. Participants indicated which panel was closer. (B) Stimulus for SD task. Participants indicated which person they would be more willing to interact and cooperate with in real life. Outside the scanner, participants rated face pairs for the level of difference between the faces in terms of the task above. (C) Results from PD task outside the scanner. Mean reaction times and standard deviations were calculated across all participants (*n* = 24). (D) Results from SD task outside the scanner.

Second, we examined whether these two aspects of ‘distance’ representation share a common neural architecture in the parietal cortex. Twenty-four healthy volunteers performed sets of tasks. First, we prepared a physical distance (PD) task and a social distance (SD) task. In the former, a display presented two inanimate objects whose relative physical positions could be inferred by texture and lighting cues, and participants indicated which object they judged to be closer to themselves (Figure 2A). In the latter, the display presented pictures of two faces, and participants indicated which individual they felt they would be more compatible with and would interact and cooperate with better in real life (Figure 2B). The facial pictures were chosen because the facial appearance of a person is known to give us the first impression of the person and if the person is attractive to us we often feel social compatibility to that person, increasing the motivation to build a sustainable relationship [19]. Then, we expect that common section in the parietal cortex is involved in the evaluation of both physical and social distance, and degree of parietal activation reflects demands of the SD task.

Next, we investigated if the parietal activation in the SD task is exclusively associated with neuronal computation for evaluating abstract ‘distance’ from other agents in the egocentric framework. We prepared another task, i.e. a social feature (SF) task, where the participants were presented with the same pairs of faces but were asked which would be more ‘socially popular’ or ‘get along with people in general’. This task replicated elements of the SD task, such as the evaluation of facial features or empathetic processing, but lacked the element of self-referential distance. Thus, it is likely that the SD task and the SF task both require common neuronal process related to the analysis of one's facial features and empathetic processing, but only the former activates the parietal cortex imposed a role of self-referential (egocentric) processing of evaluation of social distance.

Finally, we also expect that the parietal cortex communicates with other brain areas such as the fusiform gyrus, where task-relevant information about facial features is processed [e.g. 20, 21], in a way that fusiform gyrus activity, which is most likely elicited by both the SD and SF tasks, influence the parietal activity only in the SD task. We addressed these questions by measuring brain activity with a functional magnetic resonance imaging (fMRI).

## Results

### A doll-arranging task

The participants spatially arranged dolls on a stage (Figure 1A). Each participant was first asked to place a white doll (representing self) and a black doll (representing an incompatible person) wherever they liked on the stage. Next, he/she randomly picked one doll at a time out of 12 dolls (each had a facial picture of an unfamiliar person and all of the pictures were also used in the fMRI experiment), and if he/she felt that he/she would be compatible with the person in real life, he/she had to place it anywhere on the stage. Even though each participant was allowed to place the compatible dolls anywhere on the stage (Indeed, some participants simply sorted the dolls in a row fashion), the averaged distance between the self and compatible dolls across participants was significantly (physically) shorter than that between the self and incompatible dolls (paired t-test, t = 4.2, df = 14, *p*<0.001; Figure 1B). These suggest that people can choose compatible persons solely based on their facial appearance, and clearly demonstrate that people tend to spontaneously arrange representations of socially compatible individuals near themselves even without explicit instructions to do so. Thus, it is likely that people represent social relationships on a ‘distance’ scale when estimating social compatibility with other agents.

### Behavioral results in the PD and SD tasks

In order to verify the response consistency of the participants in the PD and SD tasks, all fMRI participants repeated the same PD and SD tasks outside the scanner. In the outside, in addition to the task requirements inside the scanner, the participants were also requested to provide a rating (1–5) in the SD trials for *how different* each pair of faces was in terms of the social compatibility to the participant (see methods). As social compatibility seems to be represented on a ‘distance’ scale (see above), this provided a measure of subjective social distances analogous to the objectively defined distances used in generating the PD stimuli.

We found that the reaction time (RT) became significantly longer as the differences in ‘distances’ decreased, and that this relationship was consistent across both the SD and PD tasks. That is, when pairs of faces were rated as being similar in social distance, the RTs increased (F(3, 69) = 12.2, *p*<0.005 inside the scanner, *p*<0.001 outside, single-factor ANOVA; Figure 2D). The same trend was seen in the PD task: when the two physical objects were about the same distance from the participant, the RTs increased (F(2, 46) = 43.8, *p*<0.001 inside, *p*<0.001 outside; Figure 2C). The graded RTs in the SD task imply that when people evaluate social compatibility with a person and compare these about two persons, abstract magnitudes of their social ‘distances’ could be compared, as in the case when people compare physical distances from themselves to two objects in the PD task.

Finally, the RTs for the SD task were significantly correlated inside and outside the scanner (df = 22, *r* = 0.69, *p*<0.001), indicating the consistency of the SD task demands for participants both inside and outside the scanner. Based on this finding, together with the evidence of ‘distance’-dependent processing in the SD task (Figure 2D), we used the subjective ratings of differences in social distance as covariates to depict brain regions related to this task demand (see below).

### Brain activation related to evaluation of ‘egocentric space’

Our main hypothesis was whether common section in the parietal cortex is involved in the evaluation of both physical and social distance. In order to depict brain areas related to both the PD and SD tasks, we prepared two control conditions (PC and SC, respectively). In these control conditions, participants simply pressed a button in response to the displayed objects or faces that are the same in the PD and SD tasks. Thus, by directly comparing brain activity during the tasks with that during their corresponding control conditions, we may depict activations purely related to the tasks that cannot be accounted by factors of simple visual and motor processing.

**Figure 3:**
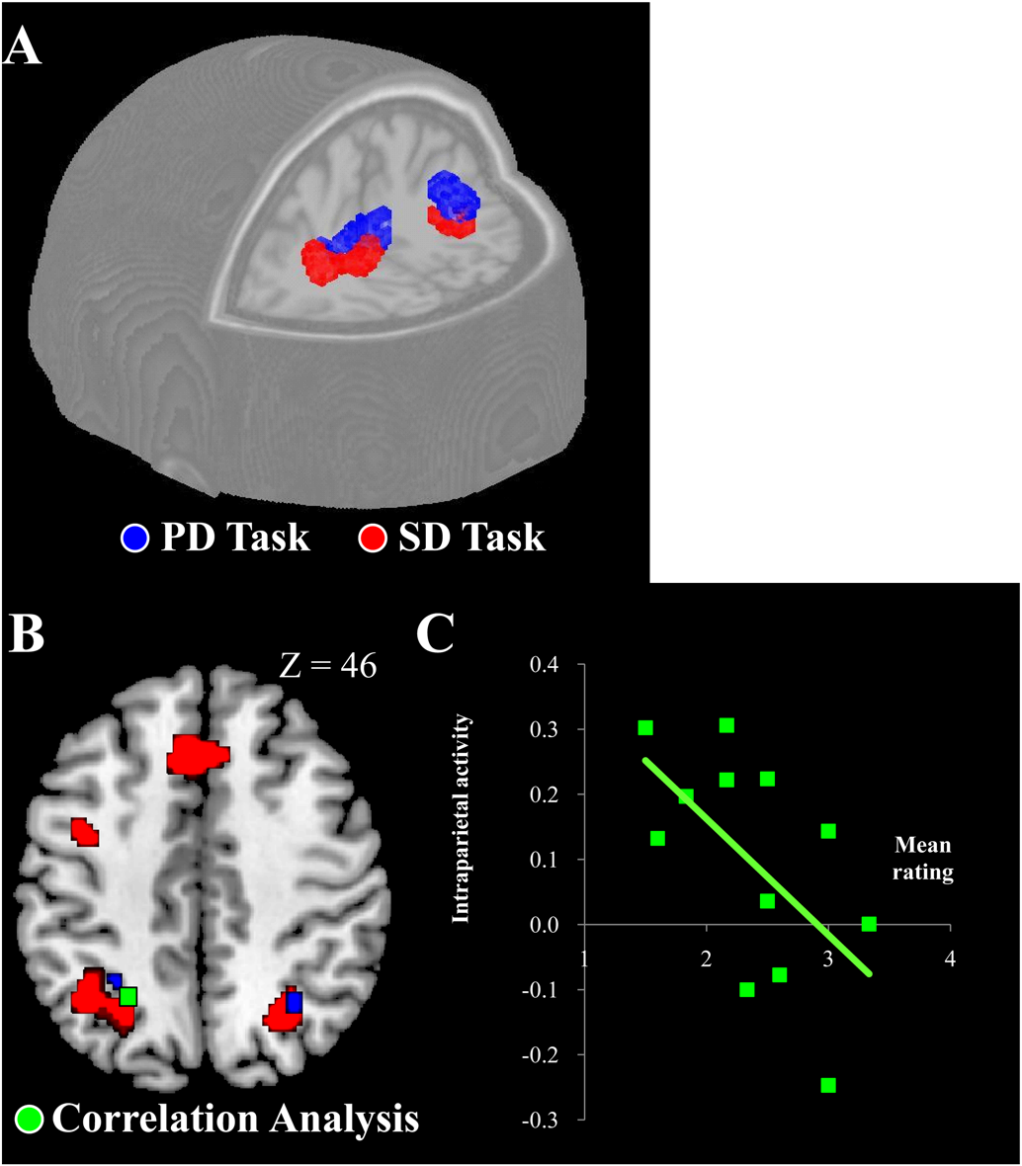
Brain activations during PD and SD, and a representative result from correlation analysis. (A) Blue sections indicate parietal activations for the PD task, identified by the contrast PD vs. PC (control task). These are the only regions identified by this contrast in the entire brain. Red regions correspond to parietal activations for the SD task (SD vs. SC). This panel illustrates only the parietal activations for the SD task. (B) In addition to areas active during the SD (red) and PD (blue) tasks, the green section in the left parietal cortex corresponds to the region whose activity was negatively correlated with the behavioural ratings in the SD task. Horizontal section *z* = +46 is displayed. (C) Negative correlation between the left intraparietal activity and the ratings in a representative participant (*N* = 12, *r* = −0.58, *p*<0.05). X-axis indicates mean ratings from 12 blocks in the SD task, and Y-axis indicates the corresponding mean level of intraparietal activity (ratio to baseline).

**Figure 4:**
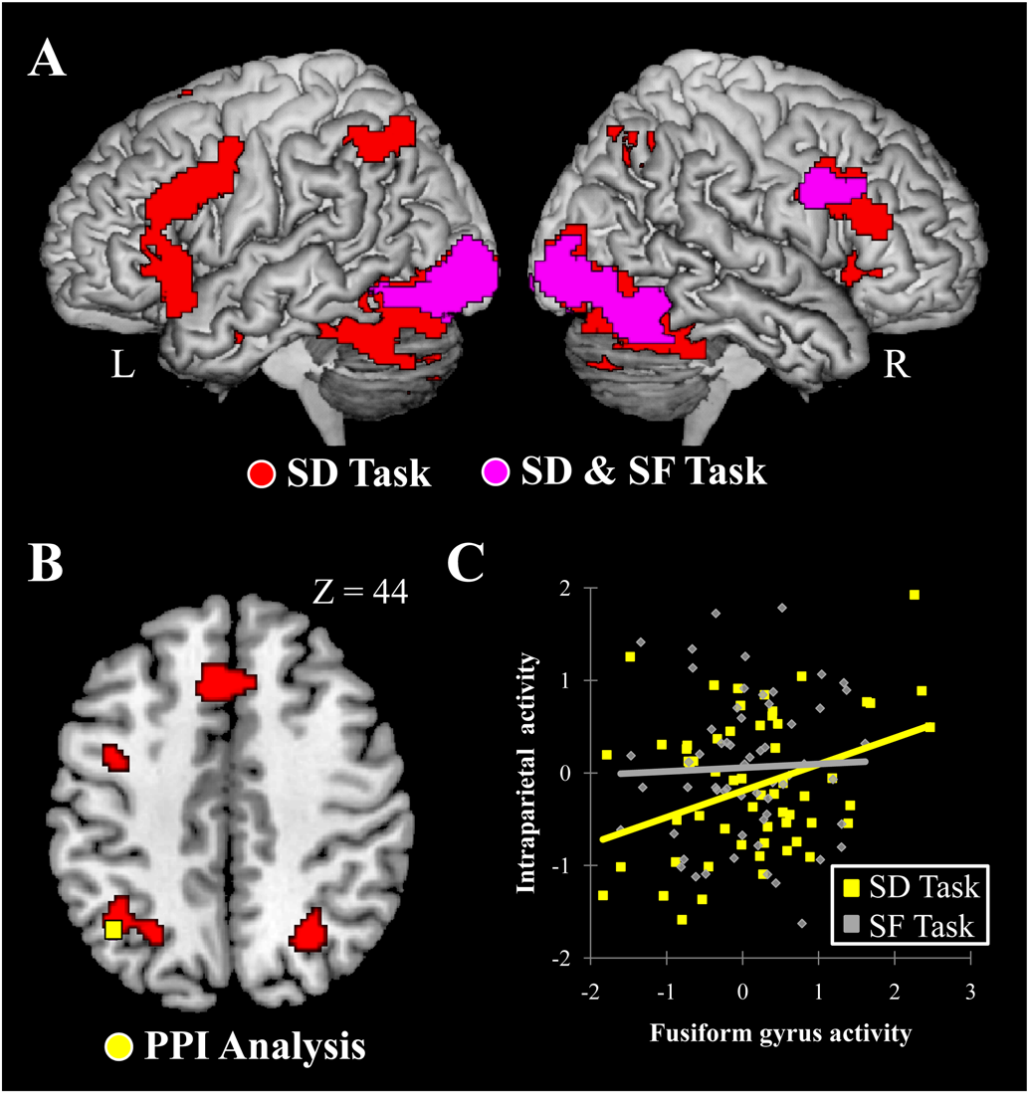
Brain activations during SD and SF (A), and results from PPI analysis (B, C). (A) Pink sections indicate brain regions active in common between in the SD and SF tasks, [Boolean intersection (SD vs. SC) ∩ (SF vs. SC) for display purpose]. Red sections indicate brain regions active during the SD task (SD vs. SC). While many regions active during the SD task were also active during the SF task, parietal activations were observed only in the SD task. The right hemisphere is displayed in the right panel. (B) The yellow section is a parietal region whose activity enhanced its coupling with that in the left fusiform region only in the SD task. Horizontal section *z* = +44 is displayed. C, Relationship between activities of the left fusiform gyrus and the left intraparietal cortex in a representative participant [*N* = 60, *r* = 0.33, *p*<0.01 for the SD task (yellow dots); *r* = 0.01, *p*>0.93 for the SF task (grey dots)]. X-axis indicates the degree of activity (mean adjusted) in each trial of the fusiform gyrus, and Y-axis indicates that of the intraparietal cortex.

The fMRI analysis revealed that the only brain activity that was significantly associated with the PD task was in the superior aspects of the posterior parietal cortices. This bilateral activation included the intraparietal regions [PD vs. PC, *p*<0.05 corrected; left and right peak coordinates, (−16, −64, 58) and (22, −68, 52); Figure 3A]. This is consistent with previous findings that implicate the parietal cortex in the estimation of egocentric distances. Notably, significant bilateral parietal activation was also found during the SD task (SD vs. SC, *p*<0.05 corrected; peak coordinates, (−38, −56, 46) and (30, −54, 38); Figure 3A), and these regions overlapped with those of the PD task (47 voxels), in a slightly ventro-lateral portion [peaks of overlapping sections, (−22, −66, 54) and (22, −70, 52)]. As expected, the SD task also activated a network of brain areas consistent with the requirements of visual face processing and general social cognition for the task: the bilateral visual cortices, extending into the fusiform gyri; bilateral medial frontal cortices; inferior frontal cortices; insular cortices; and left basal ganglia and amygdala (Figure 4A).

A more stringent method to isolate areas relevant to the SD task is to search for areas whose activities scale with the task demands. Subjective ratings of differences in social distance are a putative measure of the task demands (see above). As their validity was confirmed by the consistency and systematicity of the behavioural data (see above), we then performed parametric modulation analysis across all the brain areas activated by the SD task (Figure 3A) to find voxels whose activation correlated with the demands as measured by the social distance ratings. Within the brain areas active during SD task, only the left intraparietal cortex showed a significant correlation [peak coordinates, (−24, −60, 44); Figure 3B]. The activity in this region was negatively correlated with the social distance rating, i.e. trials in which the two faces were rated as having similar social distances resulted in greater activation of the left parietal cortex (Figure 3C).

### Comparison between Social distance evaluation and Social feature judgement

To help isolate the elements of the SD task related to egocentric social distance, we compared the activation pattern to that during the SF task. Overlapping activation for SD and SF was found in the bilateral visual cortices and fusiform gyri and in the bilateral medial frontal and right inferior frontal cortices [(SD+SF) vs. SC; see Figure 4A]. This suggests a number of areas which might underlie the cognitive functions shared between SD and SF, and some which are unique to SD. In particular, the activation of the parietal cortex appears to be unique to SD. No significant parietal activation was found in the SF task (SF vs. SC), suggesting that the common factors between SD and SF, including eye movements and attentional factors, did not contribute to the parietal activation observed only in the SD task. A direct comparison between SD and SF revealed that SD caused greater activation in the left intraparietal cortex (*p* = 0.001 uncorrected). These differences exist despite the fact that the stimuli for SD and SF were the same and that both RTs, each of which was significantly longer than that of the PD task, indicated similar difficulty levels [SF, 1597±332 ms; SD, 1768±367 ms; PD, 771±197 ms].

### Psychophysiological interaction analysis in the SD task

In our final analysis, we examined the functional connectivity of the parietal region as a measure of its relevance to the social task. If the activity in the parietal cortex actually mediates the task of assessing social distance based on the face stimuli, then we may expect that it communicates with areas where task-relevant information is processed, such as the fusiform gyrus, which is known to process facial features [e.g. 20, 21]. While both the SD and SF tasks elicited fusiform gyrus activity, that activity should influence the parietal activity only in the SD task, perhaps via the anatomical connection from the intraparietal cortex to a wide range of cerebral cortices, including the fusiform gyrus, within the same hemisphere [22], [23]. We examined such conditional coupling using psychophysiological interaction analysis [24], investigating whether the activity in the parietal cortex receives stronger contextual influences from the fusiform gyrus under the SD task compared to those under the SF task. This analysis revealed enhanced coupling in the SD task between a fusiform region (−40, −54, −26) and an intraparietal region (−40, −62, 42; Figure 4B, C) within the left hemisphere. Again, this left intraparietal region matched the region active in the SD task (*p*<0.05, after small volume correction).

## Discussion

Our results demonstrate that neuronal activity in the human parietal cortex, which is involved in the spatial processing of self-referential physical distance, seems to be associated with the evaluation of social distance between self and others. Thus, our neuroimaging finding raises a possibility that the human parietal cortex is a member of social brain network.

Only superior aspect of posterior parietal cortex including intraparietal area was activated in the entire brain when the participants assessed physical distance between themselves and external objects. This is consistent with previous notion that posterior parietal cortex is involved in self-referential coding of external objects that is often used for upcoming motor behaviors [25], [26]. The parietal lobule is often activated when people make self-referential spatial judgement of an external object, whereas the lobule becomes silent when people judge allocentric spatial location of an object [16]. Furthermore, a patient with lesion in the parietal lobule shows impairment in relating her body to external objects [15], [27]. All these findings indicate the importance of superior aspect of posterior parietal cortex in humans in a function of self-referential (egocentric) spatial processing of external world.

The SD task required the participants to evaluate social compatibility with a person based on his/her facial appearance. As demonstrated in the doll-arranging task, when people evaluate the social compatibility, they tend to think of the compatibility as a ‘distance’ that can be converted into physical distance from the self-representing doll that brought their egocentric viewpoints. Thus, it is assumed that the participants also performed self-referential (egocentric) processing of an abstract magnitude of ‘distance’ from a person in the SD task, as indicated by the graded RT in this task (Figure 2D). Then, we found activations during the SD task in the intraparietal regions that are also active during the self-referential assessment of physical distance (PD). The activations should be related to core process of the SD task because within the brain regions active during the SD task only the activity in the parietal regions reflected the demands (i.e., the differences in social distances between self and two persons) of SD task (Figure 3). Further, the activation was observed in the SD task, while the region became silent in the SF task where self-referential evaluation of a person was not particularly required, even though the brain had to process the same sets of facial pictures. Thus, it seems that self-referential (egocentric) evaluation of social compatibility with a person engages the intraparietal regions that are associated with the self-referential assessment of physical distance. In general, one should carefully interpret roles of common activations across multiple tasks. However, it might be also true that activations in the same brain region shared between tasks may indicate that the tasks commonly, at least partly, require neuronal computation implemented on neuronal machinery of the specific brain region. In this vein, egocentric evaluation of social compatibility with a person might require a brain function of self-referential assessment of ‘distance’ in the parietal cortex.

The context-dependent involvement of intraparietal cortex is further supported by the present PPI analysis. Namely, both the SD and SF tasks engaged the fusiform gyrus that plays an essential role in the facial processing, but functional coupling of activities between the fusiform gyrus and the parietal cortex in the left hemisphere was specifically enhanced in the SD task. This suggests that when the brain has to evaluate social compatibility with a person based on his/her facial appearance, the information processed in the facial processing area needs further computation for the evaluation of social distance. The parietal cortex in the dorsal pathway seems to be best situated for this neuronal computation. Thus, the findings from our analyses all suggest the predominant importance of the intraparietal cortex for the neuronal computation of the social distance evaluation, and the human parietal cortex may have social-cognitive function in spatial terms that is analogous to its intrinsic properties of spatial function.

Our ability to judge human relationship in spatial terms may have its evolutionary root in the ontologically older neural substrates for spatial processing. Converting a function of particular brain region related to an ontologically older processing of physical world into the processing for mental world could be a basic and general strategy of the brain. The present study may provide an example of this extending function in human social cognition.

In order to share a brain function between analyses of physical and mental worlds, mental quantity should be represented as abstract magnitude of it. Human parietal cortex appears also to be specialized for this purpose because parietal cortex participates in mental rotation task [28] and in the processing of magnitudes of temporal discrepancy and of numerical differences [29]–[31]. Our present findings extend the cortical network of social cognition to the parietal cortex by suggesting that the parietal cortex subserves analytic functions in evaluating social relationships [c.f. 32]. In past studies on the neural underpinning of social cognition, much focus has been given to emotional [33], [34] and reward-related brain regions [35], [36]. Growing evidence in non-human primate supports the involvement of the parietal cortex in the social evaluation. Neurons in the intraparietal sulcus exhibit activities that appear to represent values regarding other agents such as female, subordinate and dominant moneys [37]. Moreover, neuronal activities in the intraparietal sulcus are modulated in a context-dependent manner under a circumstance where ‘social’ hierarchy exists [38]. While neurons in the intraparietal sulcus are classically implicated in the spatial processing of depth [14], [39], those primitive ‘social’ functions seem to be supported by neurons in the parietal cortex. However, the most striking difference between our human observation and the monkey studies might be that humans can evaluate social distance from other agents based solely on their unfamiliar facial pictures by mentally simulating future outcomes from the cooperation with the agents.

In summary, we found activity in the parietal cortex in connection with a task involving self-referential judgments of social distance. The location of this parietal activity overlapped with areas activated during judgment of spatial distance, suggesting a shared cognitive mechanism, perhaps one of distances in the abstract. This may help explain the linguistic, psychological and behavioural connections between the concepts of the physical and social spaces. Taken together, it seems that parietal cortex may have evolved beyond its original purpose of analyzing physical space, to work as a multi-purpose module for computing abstract distances. Such a co-opting of spatial processing for the purposes of social cognition would seem useful in an evolutionary context, given the scale, complexity and abstractness of relationship networks in human societies.

## Materials and Methods

### A doll-arranging task

Fifteen participants (12 male and 3 female; ages 20–32 years) spatially arranged 6-cm-high dolls on a 30×30-cm stage (Figure 1A). Each doll had a small dot on its vertex, which was used in the distance analysis (see below). Each participant was first asked to place a white doll (representing self) and a black doll (representing an incompatible person) wherever they liked on the stage. A total of 12 dolls, each of which had a facial picture of an unfamiliar person, were tested. The pictures were selected from a publicly available facial picture database (see below), and all were also used in the fMRI experiment. The participants randomly picked one doll at a time, and if they felt that they would be compatible with the person in real life, they had to place it anywhere on the stage. All participants completed this task within 5 minutes. We took a picture from just above the final arrangement of dolls. The size of stage in the picture was edited (300×300 pixels) by a computer software, and we counted the number of pixels to measure distances between the self and compatible/incompatible dolls. When multiple compatible dolls were selected, the average distance was calculated. The doll-arranging task was a virtually modified version of the social measurement that was done to measure spatial distance between persons by carefully observing human natural social behaviors (10, 11). This task was designed to be easily done in an experimental room. Even though we have not directly tested the validity and reliability of the task in the evaluation of psychological (social) distance, together with the previous findings, the present doll-arranging task may conveniently evaluate our tendency to regulate “personal space” based on the degree of social compatibility with others.

### fMRI participants

Twenty-four healthy male (20) and female (4) volunteers (ages 19–34 years) participated in the fMRI experiment. This group did not include individuals who participated in the doll-arrangement task (see above). All participants provided written informed consent prior to the experiments, and the Ethical Committee of National Institute of Communication and Technology (NICT) approved the study. The fMRI experiment was conducted according to the principles and guidelines of the Declaration of Helsinki (1975).

### fMRI setup

A 1.5-T SHIMAZU Marconi scanner (MAGNEX ECLIPSE) with a head-coil provided T1-weighted anatomical images (3D RF-FAST) and functional T2*-weighted echoplanar images (64×64 matrix, 3.0 mm×3.0 mm, TE = 50 ms). One functional image volume of the brain was acquired every 4 s (TR = 4000 ms). A functional image volume comprised 40 slices, each 4 mm thick, which ensured that the whole brain was within the field of view (192 mm×192 mm).

Participants rested comfortably in the supine position in the scanner. Both arms were extended parallel to the trunk. In this position, the participants were able to press two buttons with their right index and middle fingers. The participants were instructed to completely relax their bodies, not to think of anything in particular, and not to make any unnecessary movements during the scanning. In the scanner, they viewed the visual stimuli, projected from outside the scanner room, through a mirror located just in front of their eyes.

### Behavioural tasks

#### Physical distance (PD) task

Participants chose which of two inanimate objects on a computer display they judged to be physically closer to themselves (Figure 2A). Each object was a flat panel with a uniform texture. We prepared four different texture panels, and displayed two of those textures in each trial. The two panels were rendered along a virtual line on the monitor (Figure 2A top); this line was tilted at three different angles (15°, 30° and 45°) to the participants' frontoparallel plane. In half the trials, the right panel was closer, and in the other half, the left. In the presentation of the stimuli, the angle of tilt (3), direction of tilt (2) and combination of textures in pairs (12) were counterbalanced. Thus, the task comprised 72 trials, which were repeated again in outside the scanner.

#### Social distance (SD) task

Participants presented with pictures of two faces chose that of the person that they would be more likely to be compatible with, as well as more willing to interact and cooperate with, in real life (see the original instructions in Japanese below). We used, with permission, facial pictures from a publicly available facial picture database produced by Softopia Japan (Gifu, Japan) for educational and research purposes. The database consists of facial (neutral) pictures of Japanese males and females across a fairly wide range of generations (ages 15–64 years). We used this database because Japanese faces would be more familiar to the participants, though the individual in the picture was unfamiliar. For the present study, we selected pictures of 36 males and 36 females ranging from 20 to 35 years of age (roughly the same generation as that of the participants). Pictures were paired off in order of age, within the same gender. Each trial stimulus comprised one of these 36 pairs, and each pair was used twice to counterbalance the left-right positions, making a total of 72 trials.

This task was also repeated outside the scanner, with an additional rating task after each trial. Participants were asked to rate the difference between the two people with respect to the main social distance task with a score ranging from 1 (small difference) to 5 (large difference). The rating was used in the correlation analysis in the fMRI experiment (see below). The mean consistency in selecting the same person across left-right reversals was 71%, and ranged from 58% to 86%.

#### Social feature (SF) task

With the same stimuli as those in the SD task, participants were asked to choose the person that would be more socially popular and would get along with people in general (see the original instruction in Japanese below). This replicates the aspects of the SD task involving the analysis of facial features and general social cognition, but lacks the element of self-referential social distance (72 trials).

#### Physical Control (PC) and Social Control (SC) tasks

The stimuli were identical to those in the PD and SD/SF tasks, respectively. Participants had to press one of the two response buttons as soon as they passively viewed the stimuli. As they repeated the PC or SC task twice, in one session the right button press was required, and in another session the left press was assigned. Thus, the button to be pressed in a session was predetermined. This order was also counterbalanced in a participant. This allowed the basic visual response and motor activity to be subtracted from the above conditions (72 trials each). The simple RTs in the PC and SC tasks were 429±123 ms and 441±137 ms, respectively, which were significantly shorter than those obtained from the PD, SD and SF tasks (p<0.001, see also text).

#### Original instructions in Japanese

The SD and SF tasks were distinguished solely by nuances in the instructions. Here, we provide the original instructions in Japanese and their translations. In the SD task, we asked 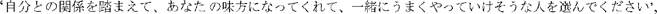 which roughly translates as ‘Given a potential relationship with yourself, choose the person who would take your side and you would get along with’. In the SF task, we asked 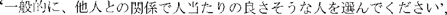 which roughly translates as ‘Choose the one who would be more accepted in human relationships, in general’.

For all tasks, stimuli were displayed for 3 s per trial, and participants were instructed to press a button as soon as they made a decision. They were also instructed to fixate on a cross displayed at the centre of the screen in order to minimize possible eye movements. Special care was taken so that the locations and sizes of paired stimuli on the monitor were identical across the PD and SD tasks.

Each of the five 72-trial tasks was split into two 36-trial sessions. Thus, the fMRI phase of the experiment consisted of 10 sessions. The purpose of the present study was to see if the parietal cortex likely active in physical distance judgment is also engaged in social distance judgment. In order to reduce confounding factors by performing these two tasks in a mixed manner, we separated the physical and social sessions. The physical (PD and PC) sessions were conducted before the social (SD, SF and SC) sessions for one half of the participants, and vice versa for the other half. Within this constraint, the order of the sessions was randomized across participants. This was done to avoid confusion of responses under the situation where the participants had to make different judgment to the identical set of visual stimuli. As both control sessions (PC and SC) were performed just before or immediately after their experimental sessions (PD, SD and SF), effect of low-frequency drift of BOLD signal could be eliminated in the contrast (e.g. PD vs. PC) even though this was not perfect.

Each 36-trial session was divided into six blocks. Each block contained six 4-s trials (a 3-s stimulus followed by a 1-s blank), for a total of 24 s (six functional images) per block. We had a period of 8 s between blocks, and this period was defined as a condition of no interest in the analysis. Thus, in each session, we collected 36 functional volumes. A total of 5 (tasks)×2 (session repetitions)×36 volumes were collected per participant.

Following the fMRI phase, participants repeated the PD and SD tasks outside the scanner using the same stimuli and timings (task and trial order were re-randomized). In the SD blocks, participants also performed the rating task described above.

### Data analysis

#### Analysis of behavioural data

The reaction times (RTs) in the PD task were sorted into three categories according to the angle of tilt used to generate the stimulus (15°, 30° and 45°), and the RTs in the SD task were sorted into four categories based on the ratings provide in the tasks performed outside the scanner (1, 2, 3 and over 4). The mean RT for each category in each task was calculated for each participant. In order to confirm that the behaviours inside and outside the scanner were consistent, the data obtained by fMRI and that obtained outside the scanner were analyzed separately, using one-factorial analysis of variance (ANOVA; repeated measurement, *n* = 24). In addition, we performed a correlation analysis across participants to see if there was a consistent trend in which participants who required longer RTs inside the scanner also required longer RTs outside the scanner.

#### fMRI data analysis

The fMRI data was analyzed with the Statistical Parametric Mapping software (SPM5; http//:www.fil.ion.ucl.ac.uk/spm; Wellcome Department of Cognitive Neurology, London). The details of general image pre-processing methods [realignment, co-registration, and normalization (MNI)] are described elsewhere [40]. The functional images were scaled to 100, spatially smoothed with an 8-mm full width at half maximum (FWHM) isotropic Gaussian kernel and temporally smoothed by a 4-s FWHM Gaussian kernel.

#### Brain activations related to PD, SD and SF tasks

A linear regression model (general linear model) was fitted to the data for each participant. Each block in a session was modelled with a boxcar function delayed by 4 s and convoluted with the standard SPM5 hemodynamic response function. We defined a linear contrast in the general linear model to identify activity that was exclusively related to the PD task by directly comparing it with activity obtained in the control task (PD vs. PC). By this procedure, we may depict brain areas that play essential roles in the PD task and are distinct from those simply related to visual processing and motor response. The same procedure was used to identify activity exclusively related to the SD task (SD vs. SC) as well as to the SF task (SF vs. SC). The participants processed identical sets of facial pictures in the SD and SF tasks; therefore, we may expect some brain regions are active in both tasks. To identify these brain regions, we tested a contrast [(SD+SF) vs. 2*SC]. Finally, as we hypothesised, we found that the PD and SD tasks activated similar regions in the parietal cortices that were not active during the SF task. Subsequently, we further tested if the parietal activations in the SD task were greater than those in the SF task by making a contrast (SD vs. SF).

The results obtained from these analyses were the estimated BOLD contrasts for each of the 24 participants (contrast images). To accommodate inter-participant variability, the contrast images from all participants were entered into a random effect group analysis (second-level analysis) [41]. A one-sample t-test was used (23 degrees of freedom). A voxel-wise threshold of *p*<0.001 (uncorrected; T>3.48) was used to generate the cluster images. For the statistical inference, we used a threshold of *p*<0.05 at the cluster level after a correction for multiple comparisons in the whole brain space.

#### Correlation analysis between rating for the social distance difference and brain activation

In the brain regions active in the SD task (SD vs. SC; Figure 3B), we looked for brain areas whose activity was correlated with the post-scan ratings for differences in social distances by performing a correlation analysis. First, we individually calculated the mean rating for each 6-trial fMRI blocks (12 blocks per participant). Then, we performed parametric modulation analysis between the ratings and effect size in the block. Effect size was obtained by comparing activity during the block with activity in the 8-s inter-block intervals. We first tested this in each participant, and then performed the random effect group analysis to accommodate inter-participant variability. In this second-level analysis, we used the contrast image (SD vs. SC) as an inclusive mask (p<0.05 corrected) to restrict the search space, which ensures that only voxels belonging to active clusters in the SD task were included. One-sample t-test was used (23 degrees of freedom). The same T-value (3.48) was used to generate the cluster images. Only the left intraparietal activity in the search space was negatively correlated with the ratings, while activity in none of searched regions was positively correlated with the ratings.

#### Psychophysiological interaction analysis

The fusiform gyrus was active in both the SD and the SF tasks. We tested if there was enhanced activity coupling between the fusiform gyrus and intraparietal cortex under the SD task, using psychophysiological interaction analysis. Since only the left intraparietal cortex was significantly correlated with the social distance ratings in the previous analysis, we focused on the data obtained from the left hemisphere for this analysis. In each participant, we extracted the time series data from a 5-mm-radius sphere around the peak (−40, −54, −26) of the left fusiform gyrus activity in common between the SD and SF tasks [(SD+SF) vs. SC]. Based on this data, a PPI regressor was computed. We constructed a linear regression model (general linear model) using the PPI regressor as well as the SD and SF regressors used in the first analysis (boxcar×hemodynamic response). Hence, this analysis was specific to the context-dependent influence of each region that occurred over and above the effects of the two tasks. For each participant, the brain regions receiving stronger contextual influences from the left fusiform gyrus under the SD task than under the SF task were tested by applying a t-contrast (1 for the SD task and −1 for the SF task). Next, the individual images were incorporated into the second-level random effect group analysis for population inference. As before, a one-sample t-test was used (23 degrees of freedom) and a voxel-wise threshold of *p*<0.001 (uncorrected; T>3.48) was used to generate the cluster images. Because of our *a priori* anatomical hypothesis, we restricted the search space and used a small volume correction [42]. The search space was defined as a 5-mm-radius sphere around the peak (−38, −56, 46) of left intraparietal activation obtained in the SD task (SD vs. SC; Figure 3B). For the statistical inference, we used a threshold of *p*<0.05 at the cluster level after the small volume correction. For the purposes of the plot illustrating activities between the left fusiform gyrus and the left intraparietal cortex in a representative participant (Figure 4C), we excluded the data from the first volume of each block (*N* = 60) to allow for the hemodynamic delay.
